# Bronchioloalveolar carcinoma as a second malignancy in a pediatric osteosarcoma survivor: case report

**DOI:** 10.1186/1477-7819-11-135

**Published:** 2013-06-12

**Authors:** Masayuki Okui, Taichiro Goto, Yuichiro Hayashi, Robert Nakayama, Mitsutomo Kohno

**Affiliations:** 1 Division of General Thoracic Surgery, Department of Surgery, School of Medicine, Keio University, Tokyo, Japan; 2 Department of Pathology, School of Medicine, Keio University, Tokyo, Japan; 3 Department of Orthopedic Surgery, School of Medicine, Keio University, Tokyo, Japan

## Abstract

**Background:**

Primary lung cancer is extremely rare in children, while secondary malignancies reportedly develop in 2% to 3% of pediatric osteosarcoma survivors.

**Case presentation:**

A 14-year-old girl was found to have two pulmonary lesions on computed tomography. These tumors had developed 1 year after osteosarcoma surgery. Segmentectomy of right segment 1 and wedge resection of right segment 9 were performed. Both lesions were completely resected and postoperative histopathological examination revealed metastasis of osteosarcoma and bronchioloalveolar carcinoma, respectively.

**Conclusion:**

Bronchioloalveolar carcinoma may present as a solitary pulmonary lesion indistinguishable from a metastatic lesion and should be included in the differential diagnosis of pulmonary lesions in survivors of pediatric cancer. Thus, pulmonary lesions identified in these patients should be biopsied or resected to establish a histological diagnosis.

## Background

Osteosarcoma accounts for approximately 3% of all pediatric malignancies. With current multi-modal approaches, 70% of patients can achieve long-term survival [1]. The lungs are a common site of recurrence, and close follow-up with imaging studies is mandatory [2]. The differential diagnosis of new lung lesions in survivors of osteosarcoma usually includes disease recurrence versus inflammatory lesions, such as granulomatous disease [3], but the possibility of a second malignancy is seldom considered. Herein, we present a surgical case of bronchioloalveolar carcinoma (BAC) found simultaneously with pulmonary metastasis of osteosarcoma. The patient was a 14-year-old girl.

## Case presentation

**Figure 1:**
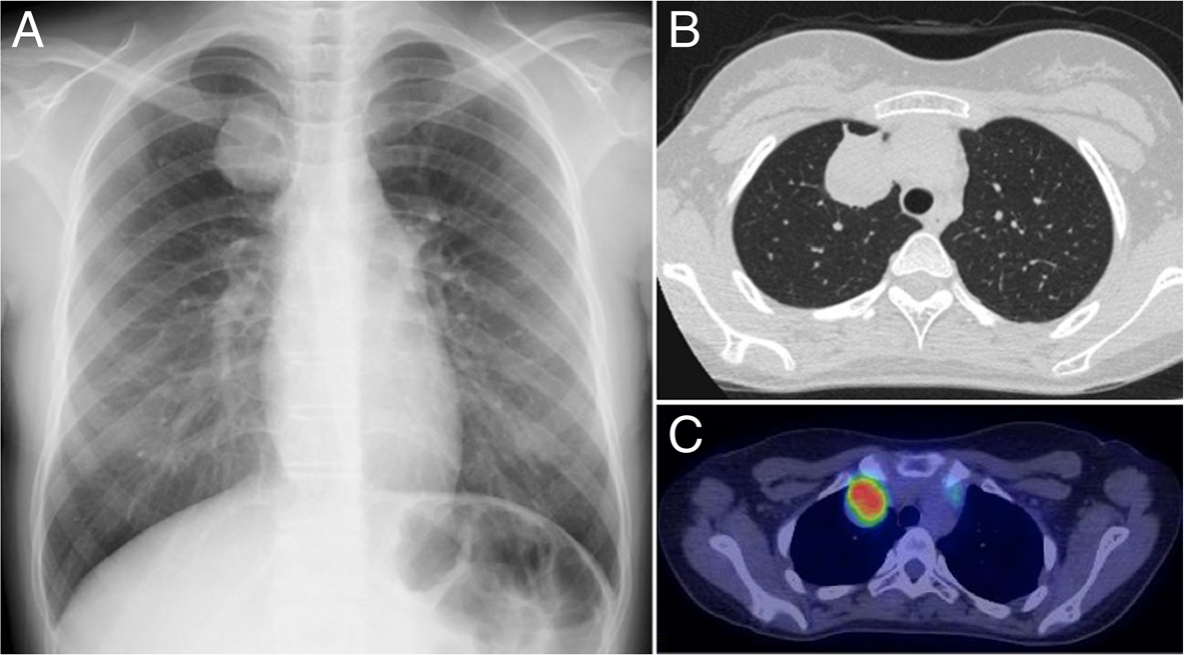
**Radiological findings of pulmonary metastasis of osteosarcoma.** (**A**) Chest X-ray shows an abnormal shadow in the right upper lung field. (**B**) Chest CT reveals a 35-mm tumor with a relatively smooth surface in right S1. (**C**) PET shows FDG uptake by the tumor.

**Figure 2:**
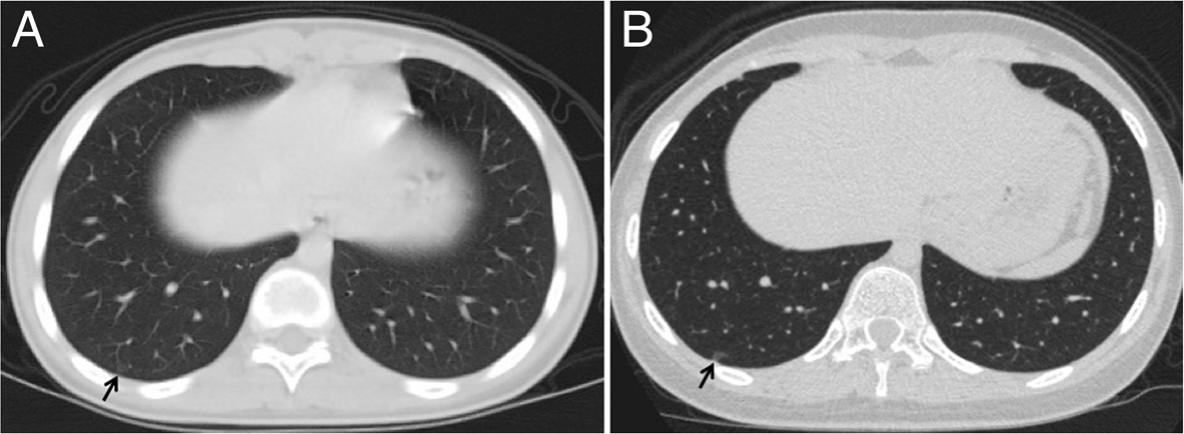
**Radiological findings of bronchioloalveolar carcinoma.** (**A**) Chest CT just before the left tibia surgery showed an extremely faint, small shadow in right S9. (**B**) When the patient was referred to our department 1 year later, chest CT revealed a 3-mm GGO at the same site.

A 14-year-old girl was found to have an abnormal shadow on a chest X-ray during postoperative follow-up for osteosarcoma of the upper end of the left tibia (Figure 1A), and was referred to our department in June 2011. Chest computed tomography (CT) showed a 35-mm tumor in right segment 1 (S1) and a 3-mm ground glass opacity (GGO) in right segment 9 (S9) (Figures 1B, 2B). Positron emission tomography (PET) showed fluorodeoxyglucose (FDG) uptake with a maximum standardized uptake value of 7.8 only in the right S1 tumor without evidence of metastatic foci elsewhere in the body (Figure 1C). Blood chemistry data were unremarkable, and the carcinoembryonic antigen and squamous cell carcinoma-related antigen were within normal limits. She was otherwise in good health. Her family history was unremarkable and negative for cancer.

As to the past history of this patient, from January 2010 to June 2010, under a diagnosis of osteosarcoma of the left tibia, she had received chemotherapy, including cisplatin + pirarubicin, high dose methotrexate, and high dose ifosfamide. Then, wide resection of the upper end of the left tibia was performed in July 2010. Although chest CT at that time showed no tumor in the right S1, a tiny and extremely faint shadow was observed in the right S9 (Figure 2A).

The right S1 tumor had a relatively smooth surface and showed rapid growth. PET showed FDG uptake, and pulmonary metastasis of osteosarcoma was suspected. On the other hand, the right S9 GGO lesion showed little tendency to grow (Figure 2), and there was no FDG uptake on PET. Given also the morphology of the lesion and the patient’s age, we considered the GGO to likely be an inflammatory lesion. At surgery, right S1 segmentectomy and wedge resection of right S9 were performed.

**Figure 3:**
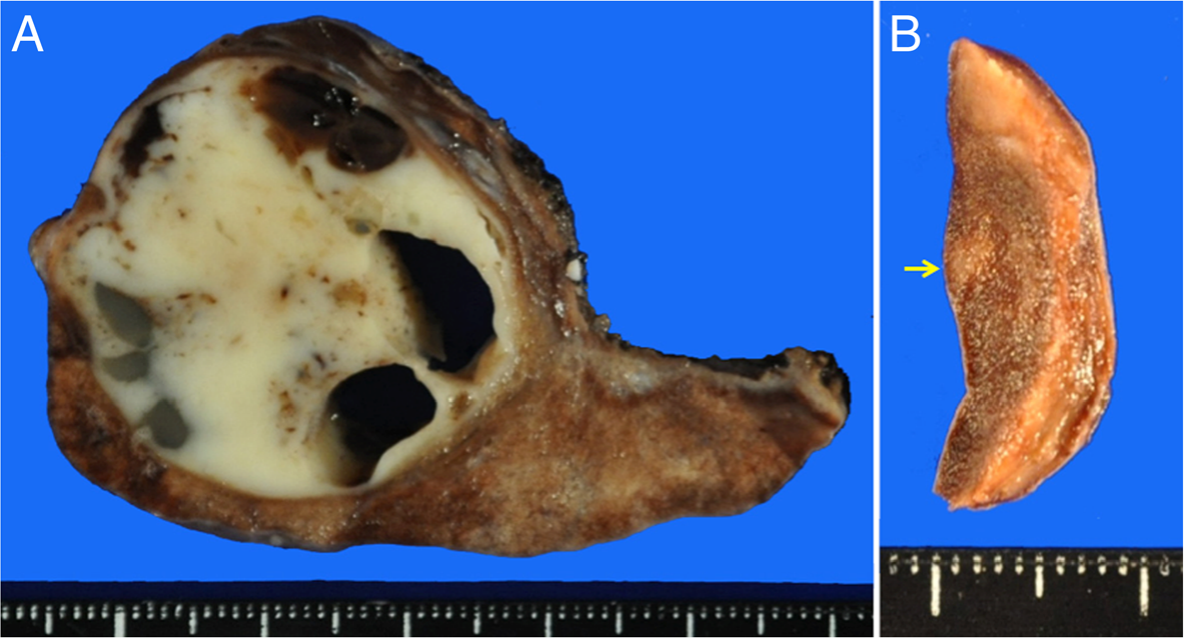
**Gross examination of the resected specimens.** (**A**) Right S1 tumor. (**B**) Right S9 lesion. Arrow indicates the S9 lesion.

**Figure 4:**
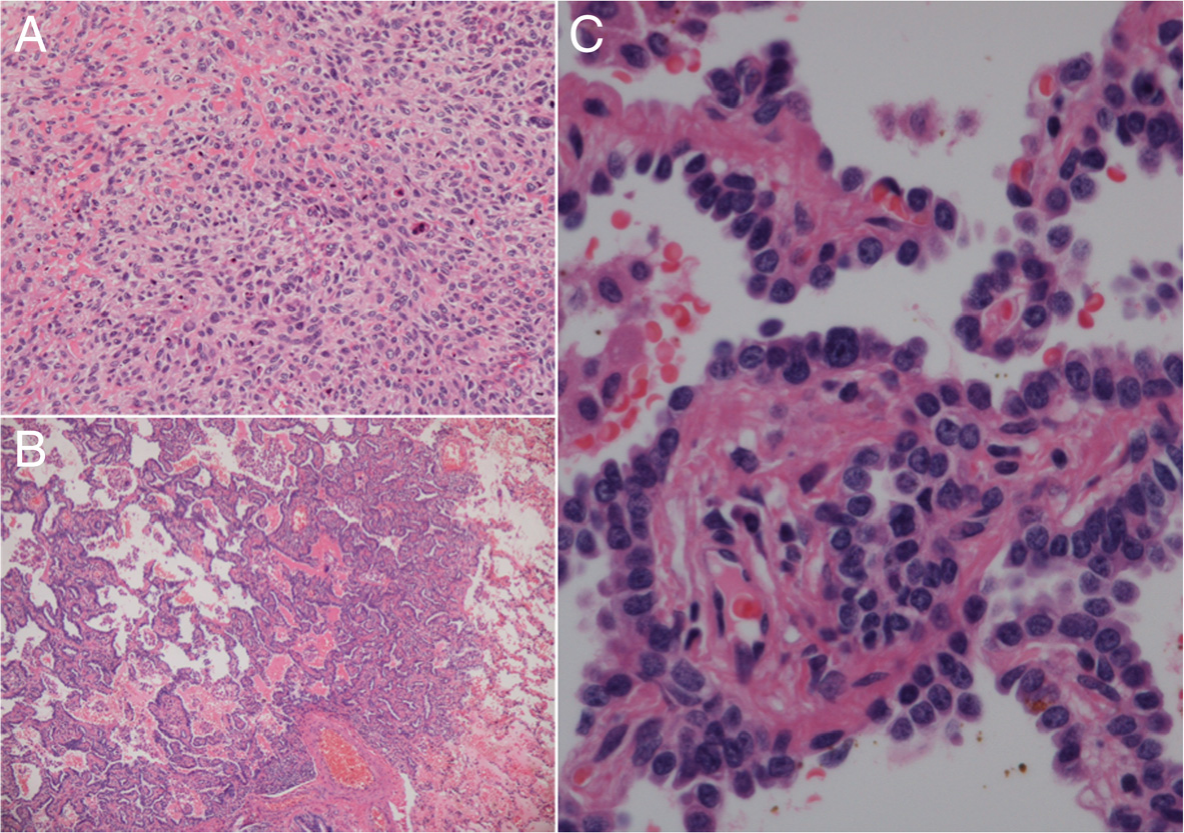
**Histopathological findings.** (**A**) Microscopic examination of the right S1 tumor confirmed a sarcoma composed of fascicular proliferation of spindle-shaped cells. (**B, C**) In the right S9 lesion, atypical cuboidal cells grew densely along alveolar walls, forming a bronchioloalveolar pattern.

The right S1 tumor was firm, with a diameter of 3.7 cm, and the cut surface was white and glistening (Figure 3A). Many cavities containing mucus were observed inside the tumor. Postoperative histopathological examination revealed spindle-shaped cells arranged densely in fascicles (Figure 4A). These cells exhibited abundant eosinophilic cytoplasm and marked nuclear pleomorphism. The tumor tissues were in part admixed with eosinophilic osteoid stroma (Figure 4A). The histology of the tumor was similar to that of the osteosarcoma resected previously. On the other hand, the right S9 lesion was an aerated whitish tumor, 3 mm in diameter with macroscopically ill-defined margins (Figure 3B). Histopathological examination revealed the alveolar walls to be densely covered with atypical cuboidal cells with enlarged nuclei, forming a bronchioloalveolar pattern (Figure 4B,C). There was no interstitial infiltration of the tumor cells. Cytologically, the cells were characterized by finely granular chromatin and conspicuous nucleoli, with atypia. Thus, the lesion was diagnosed as non-mucinous and localized BAC without foci of collapse or invasive growth. The tumor cells were positive for thyroid transcription factor-1, pan-cytokeratin, and epithelial membrane antigen. The MIB-1 labeling index was low at approximately 1%. The resected margins of both the right S1 and the right S9 tumor were free of malignant cells.

The patient had an uneventful postoperative course, and was discharged on the 7th postoperative day. Chemotherapy with high-dose ifosfamide has been performed at the Department of Orthopedics of our hospital. At present, 16 months after lung surgery, she remains recurrence free.

## Discussion

The incidence of primary pediatric lung cancers is extremely low [4]. Histologically, bronchial adenoma (40%), bronchogenic carcinoma (17%), and pleuropulmonary blastoma (16%) are the common types [4]. BAC is one such rare form of pediatric pulmonary malignancy, but no clear association between BAC and environmental exposure has been established [5]. One identified association is a link between BAC and congenital adenomatoid cystic malformation Type I, particularly with mucinous metaplasia [5-7]. Sporadic cases of BAC developing as a secondary malignancy have been reported among survivors of pediatric malignancies, including Ewing sarcoma, Hodgkin’s lymphoma, hepatoblastoma, and testicular teratocarcinoma [8-11]. As in this case, the occurrence of BAC in a child with another primary malignant tumor treated with chemotherapy or radiation therapy raises the possibility that BAC is linked to therapeutic or immunosuppressive factor, in addition to a genetic predisposition.

Although the overall incidence of second malignant neoplasms in survivors of pediatric osteosarcomas is relatively low, it is higher compared with the expected incidence of malignant neoplasm in the general population [12-14]. Institutional reviews have found the rate of second malignant neoplasms in survivors of pediatric osteosarcomas to be between 2% and 3% (Table 1) [12-14]. Pratt *et al*. reviewed 334 patients with osteosarcoma and found that second malignant neoplasms had developed in nine (2.6%) [14]. Anug *et al*. reviewed 509 patients with osteosarcoma and documented second malignant neoplasms in 14 (2.7%) [13]. No cases with primary lung neoplasms were found in either of these reviews [13,14]. In a review of 1,205 patients with osteosarcoma of the extremities, Bacci *et al*. described 26 (2.2%) who had developed a second malignant neoplasm [12]. Two of these 26 patients developed lung cancers, one adenocarcinoma and one squamous cell carcinoma at 4 and 9 years, respectively, after the osteosarcoma diagnosis. Several studies have demonstrated that female gender and being at least 10 years of age, as in our case, proved to be independent risk factors for the development of a second malignant neoplasm [12,15].

**Table 1:** Studies on second malignant neoplasms in childhood osteosarcoma survivors

**Author/Year**	**Osteosarcomas ( *n *)**	**SMN ( *n *)**	**Median interval (years)**	**Lung cancer as SMN**
Pratt [14] 1997	334	9 (2.6%)	6.3	0
Anug [13] 2002	509	14 (2.7%)	5.5	0
Bacci [12] 2006	1205	26 (2.2%)	7.6	2

*SMN*, second malignant neoplasms.

Case reports on primary lung cancer arising in survivors of pediatric osteosarcoma are shown in Table 2[16-20]. Six cases have been reported to date. In all six, the diagnosis was confirmed by surgical resection of the pulmonary tumor. The majority of these patients were at least 10 years old. There were three boys and three girls. The histological type was adenocarcinoma in all cases (including BAC in three cases).

**Table 2:** Case reports on primary lung cancer in childhood osteosarcoma survivors

**Author/Year**		**Age (years)**	**Gender**	**Primary lesion**	**Lung cancer histology**
Nonomura [19]	1994	13	Female	Right fibula	Adenocarcinoma
Kobayashi [16]	1999	14	Male	Left femur	Adenocarcinoma
Longhi [18]	2004	17	Female	Right tibia	Adenocarcinoma
Lebensburger [17]	2009	8	Male	Right fibula	BAC
Lebensburger [17]	2009	12	Female	Right fibula	BAC
Shiraishi [20]	2010	17	Male	Left humerus	BAC

Icard *et al*. reported the 5-year survival rate for stage I pulmonary lung cancer in young patients to be 70% which is not quite different from that for lung cancer in elderly patients, and that the survival rate depends on disease stage rather than age [21]. Thus, surgical resection is the treatment of choice for pediatric BAC, as for adult BAC. In our case, additional resection was not performed after wedge resection based on the pathological examination results. Given that BAC or pulmonary metastasis of osteosarcoma may occur metachronously in the future, meticulous outpatient follow-up is planned.

## Conclusion

In conclusion, we have described a very rare BAC in a 14-year-old girl with recurrent osteosarcoma. It is interesting that most of these rare pediatric BAC have occurred in children who had another malignant tumor, raising the possibility of a link between these entities. Additionally, our present patient illustrates the importance of establishing a histopathological diagnosis for suspected pulmonary metastases in children with known primary malignant tumors.

## Consent

Written informed consent was obtained from the patient for the publication of this case presentation and accompanying images. A copy of the written consent is available for review by the Editor-in-Chief of this journal.

## Abbreviations

BAC: Bronchioloalveolar carcinoma; CT: Computed tomography; FDG: Fluorodeoxyglucose; GGO: Ground glass opacity; PET: Positron emission tomography; S1: Segment 1; S9: Segment 9.

## Competing interests

The authors declare that they have no competing interests.

## Authors’ contributions

MO and TG wrote the manuscript. MO and TG performed surgery. YH carried out the pathological examination. RN and MK were involved in the final editing. All authors approved the final manuscript.

## References

[B1] Meyers PA Schwartz CL Krailo MD Healey JH Bernstein ML Betcher D Ferguson WS Gebhardt MC Goorin AM Harris M Osteosarcoma: the addition of muramyl tripeptide to chemotherapy improves overall survival–a report from the Children's Oncology Group J Clin Oncol 2008 11 633 638 10.1200/JCO.2008.14.0095 18235123

[B2] Briccoli A Rocca M Salone M Bacci G Ferrari S Balladelli A Mercuri M Resection of recurrent pulmonary metastases in patients with osteosarcoma Cancer 2005 11 1721 1725 10.1002/cncr.21369 16155943

[B3] McCarville MB Kaste SC Cain AM Goloubeva O Rao BN Pratt CB Prognostic factors and imaging patterns of recurrent pulmonary nodules after thoracotomy in children with osteosarcoma Cancer 2001 11 1170 1176 10.1002/1097-0142(20010315)91:6<1170::AID-CNCR1114>3.0.CO;2-B 11267963

[B4] Weldon CB Shamberger RC Pediatric pulmonary tumors: primary and metastatic Semin Pediatr Surg 2008 11 17 29 10.1053/j.sempedsurg.2007.10.004 18158138

[B5] Ohye RG Cohen DM Caldwell S Qualman SJ Pediatric bronchioloalveolar carcinoma: a favorable pediatric malignancy? J Pediatr Surg 1998 11 730 732 10.1016/S0022-3468(98)90200-7 9607481

[B6] Granata C Gambini C Balducci T Toma P Michelazzi A Conte M Jasonni V Bronchioloalveolar carcinoma arising in congenital cystic adenomatoid malformation in a child: a case report and review on malignancies originating in congenital cystic adenomatoid malformation Pediatr Pulmonol 1998 11 62 66 10.1002/(SICI)1099-0496(199801)25:1<62::AID-PPUL8>3.0.CO;2-Q 9475333

[B7] Kaslovsky RA Purdy S Dangman BC McKenna BJ Brien T Ilves R Bronchioloalveolar carcinoma in a child with congenital cystic adenomatoid malformation Chest 1997 11 548 551 10.1378/chest.112.2.548 9266899

[B8] Kowalski P Rodziewicz B Pejcz J Bilateral bronchioloalveolar carcinoma of the lungs in a 7 year old girl treated for Hodgkin’s disease Tumori 1989 11 449 451 2557691 10.1177/030089168907500509

[B9] Kuttesch JF Jr Wexler LH Marcus RB Fairclough D Weaver-McClure L White M Mao L Delaney TF Pratt CB Horowitz ME Kun LE Second malignancies after Ewing’s sarcoma: radiation dose-dependency of secondary sarcomas J Clin Oncol 1996 11 2818 2825 8874344 10.1200/JCO.1996.14.10.2818

[B10] Spaner SJ Raymond G Puttagunta L Bhargava R Bronchioloalveolar cell carcinoma in a child with hepatoblastoma: case report Can Assoc Radiol J 1999 11 343 345 10555511

[B11] Travis WD Linnoila RI Horowitz M Becker RL Jr Pass H Ozols R Gazdar A Pulmonary nodules resembling bronchioloalveolar carcinoma in adolescent cancer patients Mod Pathol 1988 11 372 377 2853363

[B12] Bacci G Ferrari C Longhi A Ferrari S Forni C Bacchini P Palmerini E Briccoli A Pignotti E Balladelli A Picci P Second malignant neoplasm in patients with osteosarcoma of the extremities treated with adjuvant and neoadjuvant chemotherapy J Pediatr Hematol Oncol 2006 11 774 780 10.1097/01.mph.0000243664.02174.73 17164644

[B13] Aung L Gorlick RG Shi W Thaler H Shorter NA Healey JH Huvos AG Meyers PA Second malignant neoplasms in long-term survivors of osteosarcoma: memorial sloan-kettering cancer center experience Cancer 2002 11 1728 1734 10.1002/cncr.10861 12365021

[B14] Pratt CB Meyer WH Luo X Cain AM Kaste SC Pappo AS Rao BN Fleming ID Jenkins JJ 3rd Second malignant neoplasms occuring in survivors of osteosarcoma Cancer 1997 11 960 965 10.1002/(SICI)1097-0142(19970901)80:5<960::AID-CNCR19>3.0.CO;2-U 9307198

[B15] Knowling MA Basco VE Breast cancer after treatment for osteosarcoma Med Pediatr Oncol 1986 11 51 53 10.1002/mpo.2950140112 3005815

[B16] Kobayashi H Mori T Yoshioka M Tanaka M Okuma T Iida SI Isogai M Tabira Y Kitamura N A 14-year-old boy with small primary lung cancer J Jpn Assoc Chest Surg 1999 11 144 147 10.2995/jacsurg.13.144

[B17] Lebensburger J Katzenstein H Jenkins JJ Rodriguez-Galindo C Bronchioloalveolar carcinoma as a second malignancy in osteosarcoma survivors Pediatr Blood Cancer 2009 11 499 501 10.1002/pbc.22005 19418544

[B18] Longhi A Bertoni F Bacchini P Albisinni U Mercati U Bacci G Simultaneous osteosarcoma lung metastasis and second primary lung cancer J Pediatr Hematol Oncol 2004 11 457 461 10.1097/00043426-200407000-00013 15218424

[B19] Nonomura A Mizukami Y Shimizu J Watanabe Y Kamimura R Takashima T Tsuchiya H Tomita K Simultaneous occurrence of lung adenocarcinoma and fibular osteosarcoma in a 13-year-old girl Thorac Cardiovasc Surg 1994 11 61 63 10.1055/s-2007-1016458 8184398

[B20] Shiraishi K Mori T Ohba Y Iwatani K Yoshimoto K Iyama K Three young osteosarcoma patients with small adenocarcinoma or atypical adenomatous hyperplasia of the lung Ann Thorac Cardiovasc Surg 2010 11 358 361 21030925

[B21] Icard P Regnard JF de Napoli S Rojas-Miranda A Dartevelle P Levasseur P Primary lung cancer in young patients: a study of 82 surgically treated patients Ann Thorac Surg 1992 11 99 103 10.1016/0003-4975(92)91150-8 1610262

